# Supplementary material for: HIVprotI: an integrated web based platform for prediction and design of HIV proteins inhibitors
Source: J Cheminform. 2018 Mar 9;10:12. doi: 10.1186/s13321-018-0266-y (PMC5845081; doi:10.1186/s13321-018-0266-y)
Supplement: Supplementary file 2 — Additional file 2. Source code of HIVProtI web server. [file 13321_2018_266_MOESM2_ESM.zip › HIVprotI_Source-code/hivprotI/weka-3-6-6/doc/weka/associations/tertius/IndividualInstance.html]

IndividualInstance


---


|  |  |  |
| --- | --- | --- |
| **Overview** \| **Package** \| **Class** \| **Tree** \| **Deprecated** \| **Index** \| **Help** \| **Weka's home** | |  |
| **PREV CLASS**   **NEXT CLASS** | **FRAMES**    **NO FRAMES**     **All Classes** |  |
| SUMMARY: NESTED \| FIELD \| CONSTR \| METHOD | DETAIL: FIELD \| CONSTR \| METHOD |  |


---


## weka.associations.tertius Class IndividualInstance

```
java.lang.Object
  weka.core.Instance
      weka.associations.tertius.IndividualInstance
```

**All Implemented Interfaces:**: java.io.Serializable, Copyable, RevisionHandler

---

``` public class IndividualInstance extends Instance ```

**Version:**
:   $Revision: 1.5 $

**Author:**
:   Peter A. Flach, Nicolas Lachiche

**See Also:**: Serialized Form

---

**Constructor Summary**

`IndividualInstance(IndividualInstance instance)`

`IndividualInstance(Instance individual,
Instances parts)`


| **Method Summary** | |
| --- | --- |
| `java.lang.Object` | `copy()`             Produces a shallow copy of this instance. |
| `Instances` | `getParts()` |
| `java.lang.String` | `getRevision()`             Returns the revision string. |

**Methods inherited from class weka.core.Instance**

`attribute, attributeSparse, classAttribute, classIndex, classIsMissing, classValue, dataset, deleteAttributeAt, enumerateAttributes, equalHeaders, hasMissingValue, index, insertAttributeAt, isMissing, isMissing, isMissingSparse, isMissingValue, main, mergeInstance, missingValue, numAttributes, numClasses, numValues, relationalValue, relationalValue, replaceMissingValues, setClassMissing, setClassValue, setClassValue, setDataset, setMissing, setMissing, setValue, setValue, setValue, setValue, setValueSparse, setWeight, stringValue, stringValue, toDoubleArray, toString, toString, toString, value, value, valueSparse, weight`

**Methods inherited from class java.lang.Object**

`equals, getClass, hashCode, notify, notifyAll, wait, wait, wait`

**Constructor Detail**

### IndividualInstance

```
public IndividualInstance(Instance individual,
                          Instances parts)
```

---


### IndividualInstance

```
public IndividualInstance(IndividualInstance instance)
```


**Method Detail**

### copy

```
public java.lang.Object copy()
```

:   **Description copied from class: `Instance`**
:   Produces a shallow copy of this instance. The copy has
    access to the same dataset. (if you want to make a copy
    that doesn't have access to the dataset, use
    `new Instance(instance)`

    :   **Specified by:**: `copy` in interface `Copyable` **Overrides:**: `copy` in class `Instance`
    :   **Returns:**: the shallow copy

---


### getParts

```
public Instances getParts()
```

---


### getRevision

```
public java.lang.String getRevision()
```

:   Returns the revision string.

    :   **Specified by:**: `getRevision` in interface `RevisionHandler` **Overrides:**: `getRevision` in class `Instance`
    :   **Returns:**: the revision


---


|  |  |  |
| --- | --- | --- |
| **Overview** \| **Package** \| **Class** \| **Tree** \| **Deprecated** \| **Index** \| **Help** \| **Weka's home** | |  |
| **PREV CLASS**   **NEXT CLASS** | **FRAMES**    **NO FRAMES**     **All Classes** |  |
| SUMMARY: NESTED \| FIELD \| CONSTR \| METHOD | DETAIL: FIELD \| CONSTR \| METHOD |  |


---
